# Complete mitochondrial genome of a latent wild oak tasar silkworm, *Antheraea frithi* (Lepidoptera: Saturniidae)

**DOI:** 10.1080/23802359.2017.1413290

**Published:** 2017-12-13

**Authors:** Tourangbam Shantibala, Kshetrimayum Miranda Devi, R. K. Lokeshwari, Shamurailatpam Anju, Reeta Luikham

**Affiliations:** a Animal Bioresources Division, Institute of Bioresources and Sustainable Development, Imphal, India;; b Regional Tasar Research Station, CSB, Imphal, India

**Keywords:** *Antheraea frithi*, mitogenome, sericigenous, wild silkworm

## Abstract

*Antheraea frithi* mitogenome was sequenced to understand its phylogenetic and evolutionary relationship. It comprises 22 tRNAs, 13 protein-coding genes, two rRNAs and a AT rich region. The arrangement of mitogenome gene follows the pattern of dytrysian lepidoptera. *rrnL* gene consists of two unique long consensus repeats copy. tRNAs like structure of *trnL* and *trnP* are present in *rrnL* gene and AT rich region, respectively. The phylogenetic analysis by maximum-likelihood method showed that *A. frithi* was clustered with *A. pernyi* and *A. yamamai*, the commercialized wild silkworms.

*Antheraea frithi*, the oak tasar silkworm, is an unexplored wild sericigenous insect that showed commercial potentiality in Manipur. Proper biological and genetic characterizations of *A. frithi* are essential for its successful conservation and commercialization. The complete mitochondrial genome of *A. frithi* was determined to examine its comprehensive evolutionary relationship and maternal inheritance among sericigenous groups.

Live samples of *A*. *frithi* were collected from Phaibung (1200 m ASL), Senapati district (N25°42′, E94°12′), Manipur, India. Taxonomically identified *A. frithi* was stored in the IBP, IBSD under the number WS/IBSD/008. The silk gland was used for extraction of mitochondrial DNA (mtDNA). mtDNA was extracted following the protocol of Clark and Nicklas (1970) with slight modification. The isolated mtDNA was sequenced using Illumina HiSeq 2000. PCGs of the mitogenome were identified by ORF finder. tRNA genes were identified in tRNAscan-SE Search (http://lowelab.ucsc.edu/tRNAscan-SE/). Phylogenetic trees based on maximum likelihood analysis were constructed on MEGA 5.1 Figure 1 (Tamura et al. 2011).

Mitogenome of *A. frithi* (GenBank KJ740437) was 15,338 bp in length. It contains typical composition of 37 genes found almost in mitochondrial genomes of insects, having 13 protein coding genes (PCGs), 22 *tRNA* genes, two subunit of *rrnS* and *rrnL*, and AT-rich region. The mitogenome follows orientation and gene order of ditrysian species of Lepidoptera (*trnM*, *trnI*, *trnQ*) (Chen et al. 2014). Within 13 PCGs, AT composition was maximum in the *atp8* gene (91.51%) and minimum in the *cox1* gene (72.15%). *Antheraea frithi* has high AT% (89.19%) in the control region as similar with *Artogeia melete* (89.17%) (Hong et al. 2009). *rrnL* consists of two long consensuses repeats of 42 bp and 32 bp. A unique feature of *trnL* like structure is its presence in *rrnL* gene. In the AT rich region, there is a structure with the motif ‘ATAGA’ and 19 bp poly (T) stretches at the 18 bp downstream of *rrnS* gene. *trnP*-like structure having proper anticodon but partial DHU loop is also detected.

The phylogenetic tree of *A. frithi*, built from the 13 PCGs of mitogenomes shows clustering of *A. pernyi* and *A. yamamai* in one distinct clade. Within Bombycoidea superfamily, Bombycidae, Saturniidae and Sphingoidae designated a cluster following the traditional morphology and molecular based classification (Liu et al. 2013). The superfamily, Papilionoidea forms a sister clade to Lepidopteran superfamilies (Liu et al. 2012). The study provides fundamental data useful in conservation genetics and thriving the commercial aspect among the wild silkworm diversification.

**Figure 1.**
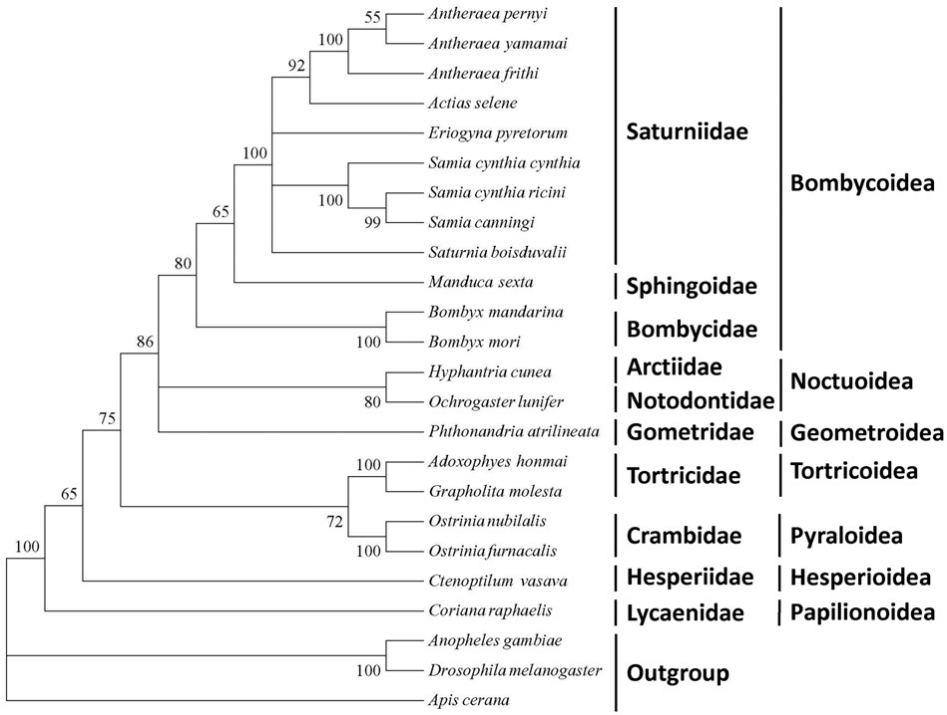
Maximum-likelihood tree of A. frithi with other related species based on 13 protein-coding genes. The sequences accession number of the species used in phylogenetic analysis are listed as follows: Antheraea pernyi (HQ264055); Antheraea yamamai (EU726630); Actias selene (NC_018133); Eriogyna pyretorum (NC_012727); Samia cynthia cynthia (NC_002084); Samai cynthia ricini (AJ400907); Samia canningi (NC_016704); Saturnia boisduvalli (NC_010613.1), Manduca sexta (NC_003368); Bombyx mandarina (NC_024270); Bombyx mori (NC_010266); Hyphantria cunea (AM946601); Ochrogaster lunifer (NC_003367); Phthonandria atrilineata (NC_007976); Adoxophyes honmai (NC_010613); Grapholita molesta (NC_014058); Ostrinia nubilalis (NC_008141); Ostrinia furnacalis (HQ116416); Ctenoptilum vasava (AB070264); Coreana raphaelis (NC_003395); Anopheles gambiae (KC812618); Drosophila melanogaster (NC_010522); Apis cerana (NC_017869).
